# Detecting cocoa plantations in Côte d’Ivoire and Ghana and their implications on protected areas

**DOI:** 10.1016/j.ecolind.2021.107863

**Published:** 2021-10

**Authors:** Itohan-Osa Abu, Zoltan Szantoi, Andreas Brink, Marine Robuchon, Michael Thiel

**Affiliations:** a Julius-Maximilians-University of Würzburg, Institute for Geography and Geology, Department of Remote Sensing, Oswald-Külpe-Weg 86, 97074 Würzburg, Germany; b European Commission, Joint Research Centre, 20127 Ispra, Italy; c Stellenbosch University, Stellenbosch 7602, South Africa

**Keywords:** Cocoa mapping, Cash crops, West Africa, Sentinel-1, Sentinel-2, Protected areas, Encroachment

## Abstract

Côte d’Ivoire and Ghana are the largest producers of cocoa in the world. In recent decades the cultivation of this crop has led to the loss of vast tracts of forest areas in both countries. Efficient and accurate methods for remotely identifying cocoa plantations are essential to the implementation of sustainable cocoa practices and for the periodic and effective monitoring of forests. In this study, a method for cocoa plantation identification was developed based on a multi-temporal stack of Sentinel-1 and Sentinel-2 images and a multi-feature Random Forest (RF) algorithm. The Normalized Difference Vegetation Index (NDVI) and second-order texture features were assessed for their importance in an RF classification, and their optimal combination was used as input variables for the RF model to identify cocoa plantations in both countries. The RF model-based cocoa map achieved 82.89% producer’s and 62.22% user’s accuracy, detecting 3.69 million hectares (Mha) and 2.15 Mha of cocoa plantations for Côte d'Ivoire and Ghana, respectively. The results demonstrate that a combination of an RF model and multi-feature classification can distinguish cocoa plantations from other land cover/use, effectively reducing feature dimensions and improving classification efficiency. The results also highlight that cocoa farms largely encroach into protected areas (PAs), as 20% of the detected cocoa plantation area is located in PAs and almost 70% of the PAs in the study area house cocoa plantations.

## Highlights

• Cocoa is a major cash crop for small-scale farmers in West Africa.
• Remotely sensed data, with open source processing, help to map such plantations.
• Cocoa farms’ role in altering complex landscapes in the region is shown.
• Cocoa farms largely encroach into protected areas (PAs), both in terms of area (1.2 Mha) and number of PAs affected (3 7 6).
• More information on PA management is needed to understand how this influences cocoa encroachment.

## 1 Introduction

*Theobroma cacao* is the only cocoa tree widely cultivated from over twenty species in the genus (Läderach et al., 2013, Nair, 2010, Peprah, 2019). Cocoa is primarily grown in parts of tropical America: Belize, Mexico, Ecuador, Peru, Costa Rica and Brazil; in West Africa: Côte d’Ivoire, Ghana, Nigeria, São Tomé and Cameroon; as well as in Indonesia (Asase et al., 2010, Attipoe et al., 2020, Kroeger et al., 2017, Malan, 2013). Cocoa represents an important component of the international commodity trade volume (Dand, 2011), providing income necessary for purchasing food for over 30 million smallholders, predominantly in developing countries (Duguma et al., 2001, Jezeer et al., 2017, Phoenix and Walter, 2009), and its production is expected to increase to meet market demand in coming years (Afoakwa, 2014, Wessel and Quist-Wessel, 2015). Côte d'Ivoire is the world's largest producer and exporter of cocoa beans, with a global market share of approximately 41%, 1650 million tonnes in 2015/16 (Friedel Hütz-Adams et al., 2016, Wessel and Quist-Wessel, 2015) and between 800,000 and 1.3 million farming households involved in cocoa production with an estimated 8 million people living off the crop. While cocoa contributed approximately 37% to Côte d’Ivoire’s exports in 2014, Ghana comes in second, with production accounting for 20% (USD 2.6 billion) of its total exports in 2016 (Hütz-Adams et al., 2016).

West Africa has lost 90% of its original moist forest and what remains is heavily fragmented and degraded (Leach and Fairhead, 2000, Salzmann and Hoelzmann, 2005). Cocoa plantations, amongst others, such as oil palm, rubber and coconut are the major drivers of deforestation in Ghana and Côte d’Ivoire (Barima et al., 2016, Smith Dumont et al., 2014). Ghana has lost more than 2.5 million hectares (Mha) (33.7%) of its forest since the early 1990 s (Oduro et al., 2015, Osei et al., 2019). The statistics for Côte d’Ivoire are even worse, as it has experienced rapid deforestation since the mid-1950 s (Leach and Fairhead, 2000). This is partially due to the increase in cocoa demand (Angelsen and Kaimowitz, 2001, Schroth and Ruf, 2014, van der Ven et al., 2018). It was recorded that between 2000 and 2013, cocoa plantations in West Africa were responsible for 57% of annual global expansion, and in 2013, 6.3 Mha was allocated to cocoa cultivation in the region (Ordway et al., 2017).

Protected areas (PAs) constitute a major conservation tool for protecting forests and the biodiversity they shelter. However, the ability of PAs to deliver positive conservation outcomes depends on how effective they are in preventing anthropogenic threats. In tropical Africa, it has been shown that the management effectiveness of several PAs is deficient, and that PAs continue to be exposed to threats such as wildlife hunting, logging and agriculture (Tranquilli et al., 2014). Cocoa farming is one of the agricultural activities threatening PAs in tropical Africa. Cocoa farmers in Côte d’Ivoire have been forced to leave their farms during civil unrest in primary forests, national parks and forest reserves (Bitty et al., 2015, Lambin and Geist, 2003, Smith et al., 2018, Yao and Roussel, 2007), and have migrated to new (forested) areas due to the search for fertile ground for the cultivation of cocoa (Hill, 1997, Ruf et al., 2015). In Côte d’Ivoire, more than one million people live in PAs, attracted by the possibilities of earning an income through natural resource extraction activities in them (Lindsey et al., 2013, Watson et al., 2014). Bitty et al. (2015) surveyed 23 PAs in Côte d’Ivoire between 2010 and 2013 and found cocoa plantations in 20 of them, representing 74% of the total surface of the PAs surveyed. They also found that 16 PAs exhibited a degradation of their forest habitat exceeding 65% and that most of this degradation resulted from cocoa farming. Buffer zones around PAs with varying amounts of human activity, including cocoa agroforestry, have been suggested as a management strategy to reduce the influence of surrounding land-use activity on PA biodiversity in Côte d’Ivoire and Ghana (Asare et al., 2014, Bitty et al., 2015). However, despite the fact that cocoa farming in PAs has been documented for a subset of PAs in Côte d’Ivoire and that cocoa agroforestry around PAs has been proposed as a mitigating solution, the lack of a comprehensive cocoa map in these two countries, the world's largest cocoa producers, has prevented any comprehensive assessment of cocoa encroachment into and around PAs thus far. This paper fills that gap.

Cocoa farm areas have been estimated by the FAO based on surveys and expert estimates from FAO member states (FAOSTAT); however, the accuracy of the FAO dataset is not reported. The FAOSTAT (2018) area estimate provides figures based on the national level for both countries. However, an accurate map of cocoa plantations is missing. Such a map could not only provide area estimates, but also reveal the actual location of existing and new plantations in both countries. Remote sensing techniques have proven to be useful in mapping crops and in the estimation of farm areas (Kuenzer and Knauer, 2013, Sun et al., 2019, Wang et al., 2019; Wójtowicz et al., 2010); the use of satellite imagery has become sufficiently accurate and operational in recent years (Jianya et al., 2008, McRoberts et al., 2010, Sun et al., 2019, Wójtowicz et al., 2010). However, cocoa farms can hardly be separated from other vegetation cover like natural forest or rubber fields based on their spectral signatures alone (Asubonteng, 2007). The use of Synthetic Aperture Radar (SAR) technologies which penetrate clouds, provides season-independent information about land surface features, including plant morphology (Lavreniuk et al., 2017, McNairn and Shang, 2016, Wang et al., 2014). The combination of optical and radar data derivatives have proven to be useful in detecting shrub crops grown under forest canopies (De Alban et al., 2018). Texture analysis can also be used to differentiate cocoa farms from rubber, oil palm and other tree types (Descals et al., 2019, Numbisi et al., 2019). Combining such features with a Random Forest (RF) classification model (Breiman, 1999), farms have successfully been used to map cocoa on a small scale. Numbisi et al. (2019) noted that the level of confusion between cocoa agroforests and transition forests was low compared to other classes. This indicates that optimising the image texture information improved the classification and helped to identify vegetation classes with a highly heterogeneous canopy.

The objectives of this study are to (1) generate and present a cocoa thematic map for Côte d’Ivoire and Ghana for the year 2019 using a state of the art cloud-based computing platform and free and open access satellite data; (2) estimate the area of cocoa plantations based on the developed thematic maps; and (3) assess the extent of cocoa farming within and around protected areas.

## 2 Materials and methods

### 2.1 Study area

**Fig. 1:**
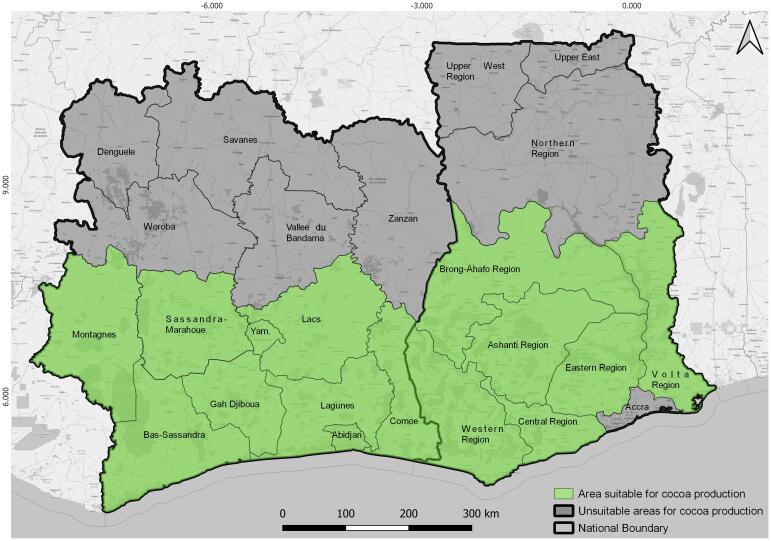
The climatically most suitable cocoa regions in Ghana and districts in Côte d’Ivoire (Läderach et al., 2013).

An approximate 20°belt either side of the Equator with humid tropical climates with regular rain and a short dry season is favourable to the growth of cocoa (Kyei et al., 2011). The trees need even temperatures between 21 and 23 °C, with a fairly constant rainfall of 1000–2500 mm per year (cocoa-growing countries). As shown in Fig. 1, climatically the most suitable cocoa areas in Ghana (GH) are mainly in the Eastern, Central, Ashanti, Western and southern Brong-Ahafo regions, while in Côte d'Ivoire (CIV) they are mainly in Sud-Comoé (Comoé since 2011), Agnéby (Lagunes since 2011), Moyen Comoé (Comoé since 2011) and Sud-Bandama (Bas-Sassandra since 2011) districts (Läderach et al., 2013). Accordingly, we focused our mapping efforts on these regions and districts and some others neighbouring them (Volta (GH), Abidjan (CIV), Gôh-Djiboua (CIV), Lacs (CIV), Montagnes (CIV), Sassandra-Marahoué (CIV) and Yamoussoukro (CIV)).

#### 2.1.1 Côte d’Ivoire

Côte d’Ivoire (latitude 8°00 N, longitude 5°00 W) borders Ghana to the east, Liberia to the west, Burkina Faso to the northeast, Guinea and Mali to the northwest and joins a 515 km coastline to the Côte Gulf of Guinea, fringed by a network of large lagoons. It occupies a land mass of 322,463 square kilometres. The climate in northern Côte d’Ivoire is characterised by lower annual rainfall which is less than 900 mm and by a high rate of potential evapotranspiration due to the high temperatures throughout the year with generally mid-range temperatures between 20 °C and 30 °C (Walz et al., 2015). Southern Côte d’Ivoire is characterised by a rainy season that lasts from April to October with high rainfall which ranges from 1000 mm to 2400 mm and a dry season that lasts from November to March (Walz et al., 2015).

Côte d’Ivoire has three vegetation zones, mainly Guinea savannah, forest savannah and tropical rainforest. The Guinea savannah zone is characterised by woodland, fire-resistant short trees and tall grass (Walz et al., 2015). The forest savannah zone is the transition zone between the Guinea savannah and the tropical rainforest, which is highly modified by anthropogenic land use due to logging and extensive farming activities (Walz et al., 2015). The tropical rainforest is characterised by an evergreen or semi-evergreen rainforest and exhibits one of the world́s biodiversity hotspots (Klopper et al., 2002, Walz et al., 2015).

Côte d’Ivoire has predominantly flat to undulating plains, plateaus, mountains in the northwest which are part of the Fouta Djallon Highlands. The rivers, Sassandra, Bandama and Comoé, traverse southern Ivorian floodplains which have very productive soils.

#### 2.1.2 Ghana

Ghana lies between latitudes 4°30′N–12°00′N and longitudes 1°12′E–3°15′W in West Africa and borders the Gulf of Guinea, between Côte d'Ivoire and Togo. It occupies a land mass of 238,535 square kilometres. The mean annual rainfall during this period is between 800 mm and 1100 mm (Abdulai et al., 2018, Asante et al., 2017, Schroth and Ruf, 2014). The rainfall is erratic spatially and in duration. There is a long dry season from November to mid-February, characterised by cold, dry and dusty harmattan winds. Temperatures during this period can be as low as 14 °C at night, but rise to more than 35 °C during the daytime (Asante et al., 2017, Schroth and Ruf, 2014).

Ghana is primarily composed of low plains with a dissected plateau in the south-central area. Wooded Guinean savannahs are characteristic of southern Ghana, which consist of the rainforest, semi-deciduous and the transition zones (Hall and Swaine, 2013). The south-eastern rugged mountain chain has lush forested vegetation cover (Hall and Swaine, 2013). Sudan savannahs characterise northern Ghana with a dry climate, open canopy savannahs and irrigated and rain-fed croplands (Hall and Swaine, 2013). The country is divided into two main agro-ecological zones: the northern savannah and the southern forest zone. The savannah zone includes the Sudan, Guinea and the coastal zones, while the southern forest region consists of the rainforest, semi-deciduous and the transition zones (Hall and Swaine, 2013).

### 2.2 Remotely sensed data

This study uses Sentinel-1 and Sentinel-2 images of all the months of the year of 2019. They are available in Google Earth Engine (GEE) as image collections. Sentinel-1 (S1) is a Synthetic Aperture Radar (SAR) system, which provides dual polarisation data at 10 m spatial resolution every 6 days (Geudtner et al., 2014, Geudtner et al., 2013). The initial data were Ground Range Detected (GRD) scenes provided as calibrated and orthorectified products (Gorelick et al., 2017). In this study, Vertical transmit/Horizontal receive (VH) and Vertical transmit/Vertical receive (VV) dual polarisation data acquired in Interferometric Wide swath mode were used.

Sentinel-2 (S2) is a multispectral optical imaging mission, which provides optical data with 13 bands every 5 days (2 satellites). The bands have spatial resolutions varying from 10, 20 and 60 m (Drusch et al., 2012). The Sentinel-2 data were provided as the top of atmosphere reflectance level (Level 1C) (Gorelick et al., 2017). The cloud interference on the images was filtered with a 6% threshold over the full scenes in GEE. To mask out certain land cover/uses for 2019, we employed two global datasets, also available in GEE – the JRC Global Surface Water Mapping Layers, v1.2 (Pekel et al., 2016) and the Global Human Settlement Layers, Built-Up Grid (Pesaresi et al., 2015) – and also had access to the Global Closed-canopy Oil Palm Plantations dataset (Descals et al., 2021).

### 2.3 Training and validation data

The ground control dataset collected in Ghana and Côte d’Ivoire is based on 19,196 points consisting of cocoa, rubber, shrubland and closed forest. Points were used instead of polygons because GEE processes polygons by converting them into spatial points and care has to be taken to create similar-sized polygons for strong autocorrelation between pixels of the same polygon. We had access to 3,842 cocoa field points collected in Ghana and 973 cocoa field points from Cocoa Life (Interactive Farm Map) for both countries. Stratified random sampling was performed on the Copernicus Global Land Cover map (CGLS-LC100) (Buchhorn et al., 2020) to extract 9272 shrub points and 2889 points for the closed forest class. Each of the collected points was assessed for equal distribution and full coverage of the assigned class variable. Additionally, 2220 rubber points were collected via visual inspection from GoogleEarth Pro, since rubber plantations can be clearly identified in very high resolution satellite images. The points were subdivided for cross-validation (90% of the points for training and 10% of the points for validation), i.e. 17,277 for training and 1919 for validation. The image pixels at each point location were then used as training in the classification. Overall accuracy (OA), producer's accuracy (PA) and user's accuracy (UA) were used as the accuracy metrics (Congalton, 1991).

### 2.4 Workflow

**Fig. 2:**
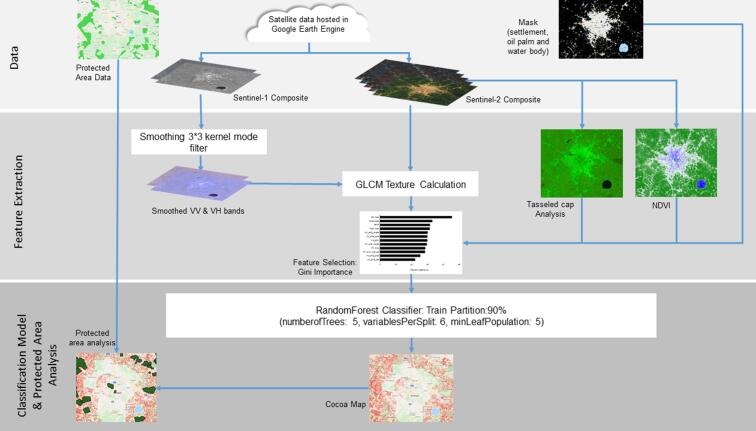
Satellite data processing workflow for cocoa plantation mapping.

The processing chain (Fig. 2) contains Sentinel-1 image composites and Sentinel-2 cloud-free image composites and derivatives generated in GEE. Settlements, water bodies and oil palm were masked out and features were selected to extract cocoa farms using the Smile Random Forest (RF) classifier in GEE.

#### 2.4.1 Satellite data pre-processing

GEE uses the steps implemented by the Sentinel-1 Toolbox to derive the sigma nought (σ°) backscatter coefficient (ESA, 2019, Gorelick et al., 2017, Gulácsi and Kovács, 2020, Veci et al., 2014). The S-1 Toolbox processing steps included: apply orbit file, GRD border noise removal, thermal noise removal, radiometric calibration and terrain correction (orthorectification), and have been completed in GEE (ESA, 2019, Gulácsi and Kovács, 2020; Sentinel-1 Algorithms | Google Earth Engine; Veci et al., 2014). Sentinel-1 data was filtered to get images with both VV co-polarised and VH cross-polarised bands. We applied a median filter with a smoothing 3 × 3 kernel mode filter to reduce the inherent speckle noise of the pre-processed S-1 images. This was done to improve the quality of S-1 images and make it ready for land use land cover classification (Khan et al., 2020; Sentinel-1 Algorithms | Google Earth Engine).

The dataset consists of median composites of S-1 (VV and VH) and S-2 (13 bands) for the year 2019 (Fig. 2). The size of the median window was set to the twelve-month period lasting from 1/1/2019 to 31/12/2019 because it was the minimum window size that could produce a cloud-free composite for S-2 in the study area. After initiating these steps, the S-1 composite was created for deriving S-1 derivatives and layer stacking with S-2 composite’s derivatives for the classification. Based on the S-2 composite, the NDVI calculation and Tasseled Cap Analysis (TCA) has been performed using functions written in GEE. Oil palm plantations (Descals et al., 2021), settlements (JRC Global Human Settlement Layer) and water bodies (JRC Global Surface Water Explorer) were masked out from the S-1 and S-2 composite and the derivatives.

#### 2.4.2 Feature extraction and selection

Feature extraction and selection is widely used to reduce the feature space and thus the data load for the modelling process. It is also reported that reducing the feature space can improve the quality of the classification (Dash and Liu, 1997, Löw et al., 2013). Thus, feature extraction aimed to select the set of most informative features from the S-1 and S-2 composites. We derived median features calculated on a 3 × 3 window size and texture features based on the gray-level co-occurrence matrix (GLCM) (Conners et al., 1984, Haralick et al., 1973). The GLCM features were derived using a 3 × 3 window size, in all directions; this was performed to capture the characteristics of very small scale cocoa farms in a fragmented landscape. The resulting output of the GLCM features was 254 texture bands (Table 1).

**Table 1:** S-1 and S-2 features derived in this research. Vertical transmit/horizontal receive (VH) and vertical transmit/vertical receive (VV) are the cross-polarisation bands of Sentinel-1. Bx represents the 13 Sentinel-2 spectral bands.

Operation	Abbreviation	Input Bands
Median	VV_sm2, VH_sm2	VV, VH
TCA Brightness	Brightness	Bx
TCA Wetness	Wetness	Bx
TCA Greenness	Greenness	Bx
GLCM asymmetry	asm	VV_sm2, VH_sm2, Bx
GLCM contrast	contrast	“
GLCM correlation	corr	“
GLCM variance	var	“
GLCM inverse difference moment	idm	“
GLCM sum average	savg	“
GLCM sum variance	svar	“
GLCM sum entropy	sent	“
GLCM entropy	ent	“
GLCM difference variance	dvar	“
GLCM difference entropy	dent	“
GLCM correlation 1	imcorr1	“
GLCM correlation 2	imcorr2	“
GLCM max. correlation	coef. maxcorr	“
GLCM dissimilarity	diss	“
GLCM inertia	inertia	“
GLCM cluster shade	shade	“
GLCM cluster prominence	prom	“

The Normalized Difference Vegetation Index (NDVI) is an important vegetation index as seasonal and inter-annual changes in vegetation growth and activity can be monitored (Weiss et al., 2004, Wu et al., 2014). The dynamic range of the NDVI is an indicator for high and low biomass. NDVI values are high in forested areas and low in areas with little or no vegetation (Zaitunah et al., 2018, Zhang et al., 2014).

Tasseled Cap Analysis (TCA) incorporates more information into vegetation indices by using six different bands (Crist, 1985, Crist and Cicone, 1984, Healey et al., 2005). The first TCA band corresponds to the brightness of the image, the second band the greenness and the third the wetness.

To rank the most relevant features for the classification model, a total of 260 features from texture features, the NDVI, Tasseled Cap features and the median filter of VV and VH bands of S-1 were analysed with Gini variable importance. Gini is the total decrease in node impurity averaged over all decision trees of the Random Forest (Descals et al., 2019, Han et al., 2016). The Gini coefficient was implemented in the Scikit-learn library (Pedregosa et al., 2011) and measures the importance of the input feature with respect to the class (Bisong, 2019). The Gini coefficient is calculated as the total decrease in node impurity averaged over all decision trees of the Random Forest (Deschamps et al., 2012, Han et al., 2016). Each feature is ranked based on the contribution it makes to the RF model (Descals et al., 2019, Genuer et al., 2010). The higher the Gini feature importance values, the more important the features in the RF model (Altmann et al., 2010, Cutler et al., 2007). The features that had an importance level greater than 0.25 were used in the RF classification model. The first ten important bands were selected based on Gini variable importance to improve the RF model.

**Fig. 3:**
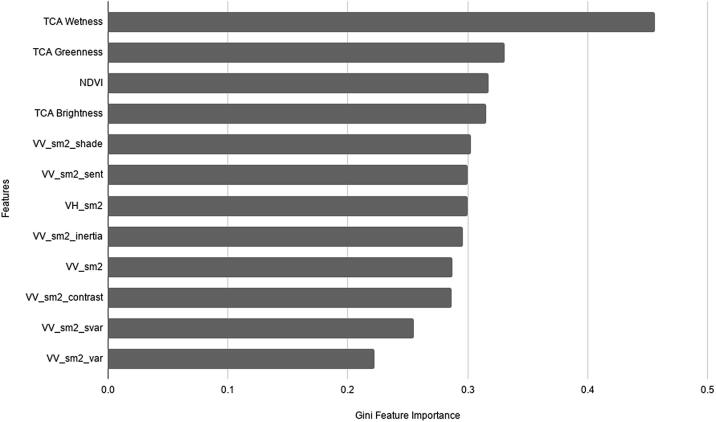
Relevant bands by Gini feature selection from the Random Forest classification. Abbreviations: VV - vertical transmit/vertical receive; VH - vertical transmit/horizontal receive dual polarisation radar data; sm2 - median filter (3 × 3 window size); shade - cluster shade; sent - sum entropy; svar - sum variance.

Fig. 3 presents the most relevant bands for the classifier based on their Gini importance evaluation.

#### 2.4.3 Classification

The RF algorithm was trained over the selected set of S-1 and S-2 features (Fig. 3) using the ground control dataset. The image classification was performed using the RF classifier in GEE. In order to identify the optimal RF classification parameters – number of trees (ntree), number of variables (mtry) and leaf population (lpop) – various combinations of parameter settings were tested against the user's and producer's accuracy of the cocoa class. We found the best settings to be 5 for ntree, 6 for mtry and 5 for lpop. Prior to the classification, 90% of the sample points were selected and used to train the RF classifier, while the remaining 10% were reserved for testing the accuracy of the classifier.

#### 2.4.4 Analysis of cocoa encroachment within and around protected areas

Data on protected area locations and their boundaries were obtained from the World Database on Protected Areas (WDPA, accessed 22 February 2020), managed by the United Nations Environment Programme World Conservation Monitoring Centre (UNEP-WCMC) with support from the International Union for Conservation of Nature. The IUCN classifies protected areas (PAs) based on their management categories, as shown in Table 2. As the reporting of PA management categories is a voluntary process from governments to UNEP-WCMC (Dudley et al., 2013), not all PAs can be assigned to a management category. The management category is not reported for an overwhelming majority of PAs in Côte d’Ivoire, while the dominant category for PAs in Ghana is category VI – corresponding to traditional natural resource management systems (Table 2).

**Table 2:** Number of protected areas (PAs) per IUCN management category in Côte d’Ivoire (CIV) and Ghana (GH).

IUCN Management Category	Description	Number of PAs in CIV	Number of PAs in GH
Ia	Strict Nature Reserve, human visitation, use and impacts are strictly controlled.	2	1
II	National Park, protects natural biodiversity and large-scale ecological processes.	8	8
III	Protected areas set aside to protect a specific natural monument.	0	57
IV	Habitat/Species Management Area, protects particular species and habitats.	1	80
V	A protected area where the interaction of people and nature over time has produced an area of distinct character.	0	6
VI	Protected area with sustainable use of natural resources, conserve ecosystems and habitats together with associated cultural values and traditional natural resource management systems.	0	122
Not reported	Management category has not been reported.	224	7
Not Applicable	The IUCN management categories are not applicable to a specific national designation type.	4	1
Total		239	282

Furthermore, to evaluate the encroachment of cocoa plantations in PAs, we overlaid the cocoa maps with the map of PAs to calculate the area of cocoa plantations within each PA. We also calculated the area of cocoa plantations within the 5 km internal buffer, the 5 km external buffer and the 10 km external buffer of each PA. Finally, we compared our results regarding the presence and area of cocoa plantations to those of Bitty et al. (2015) for the subset of the same 23 PAs for which they estimated cocoa areas based on ground survey data collected between 2010 and 2013.

## 3 Results and discussion

### 3.1 Map accuracy and plantation area estimation

**Fig. 4:**
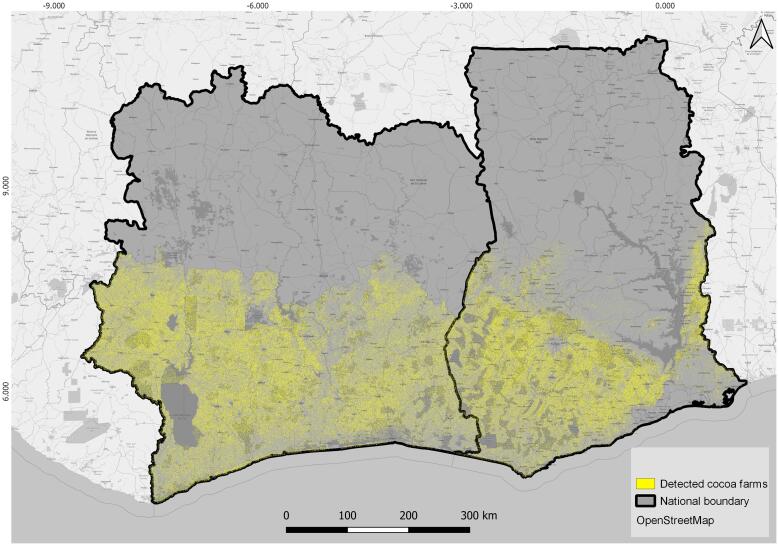
Cocoa plantations map for Côte d’Ivoire and Ghana representing the year 2019.

The cocoa plantation thematic map was obtained using the optimal Random Forest classifier parameters with selected features (Fig. 3). The producer's accuracy in mapping cocoa farms was 82.9% and the user's accuracy was 62.2%. The cultivated cocoa area (Fig. 4) occupies approximately 3.69 Mha in Côte d’Ivoire and approximately 2.15 Mha in Ghana. The map (Fig. 4) shows the small-scale nature of cocoa plantations, highlighted by the fragmented, ‘salt and pepper’-type distribution of cocoa plantations.

According to FAOSTAT, Côte d’Ivoire’s cocoa farms cover approximately 4 Mha, which corresponds to 12.4% of the land area. FAOSTAT recorded 1.78 Mha for Ghana, approximately 7.5% of its land area. Our estimates show a 1.51% difference in Ghana and −0.97% difference in Côte d’Ivoire (Table 3). The difference in area estimate may be explained by an increase in cocoa area when compared to FAOSTAT 2018, but also by the different assessment methodologies (Alexandratos, 2005; Dixon et al., 2001, Godoy, 1992).

**Table 3:** Total area of cocoa plantations mapped in Côte d’Ivoire and Ghana in 2019.

Country	District/Region	Area of cocoa in million hectares	Area of cocoa in percentage	Total land area in MHa
Côte d’Ivoire		3.69	11.4%	32.24
	Abidjan	0.03		
	Bas-Sassandra	0.56		
	Comoe	0.26		
	Gah-Djiboua	0.44		
	Lacs	0.45		
	Lagunes	0.46		
	Montagnes	0.79		
	Sassandra-Marahue	0.66		
	Yamoussoukro	0.04		

Ghana		2.15	9%	23.85
	Ashanti	0.53		
	Brong-Ahafo	0.33		
	Central	0.22		
	Eastern	0.37		
	Volta	0.20		
	Western	0.51		

### 3.2 Cocoa plantations in various districts/regions in the study area

According to the climatically most suitable cocoa areas (see Fig. 1), six regions in Ghana (Ashanti, Brong-Ahafo, Central, Eastern, Volta and Western) and nine districts in Côte d'Ivoire (Abidjan, Bas-Sassandra, Comoé, Gôh-Djiboua, Lacs, Lagunes, Montagnes, Sassandra-Marahoué, Yamoussoukro) were mapped and evaluated for their cocoa plantations.

**Fig. 5:**
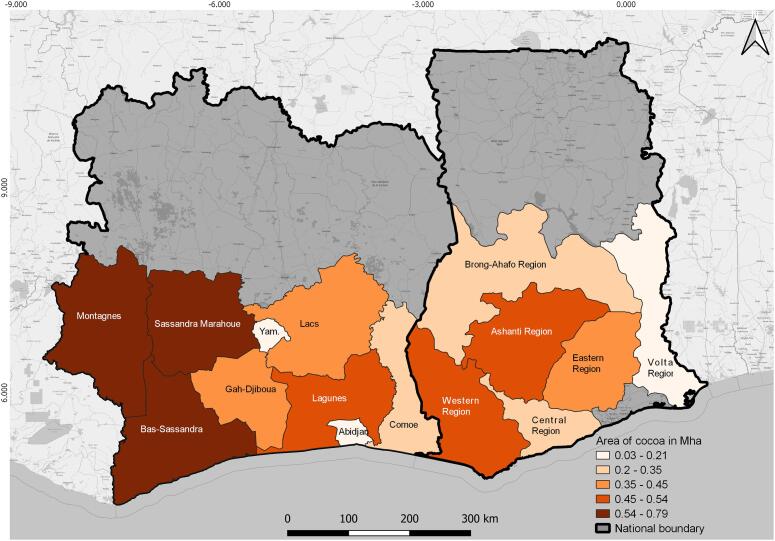
Cocoa area in million hectares (Mha) in various districts in Côte d’Ivoire and regions in Ghana.

**Fig. 6:**
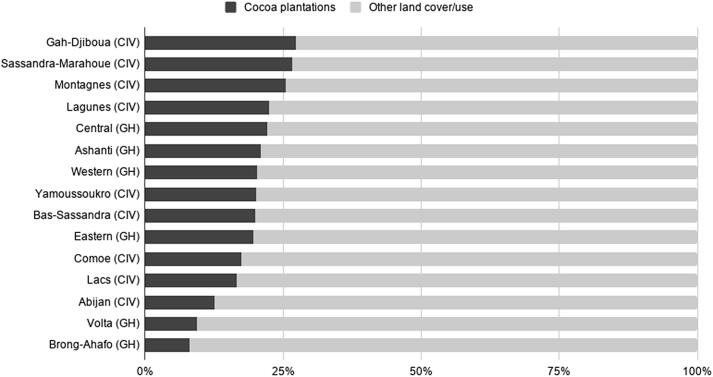
Percentage of cocoa plantations in Ivorian districts and Ghanaian regions.

We detected 3.69 Mha of cocoa plantations in Côte d'Ivoire. The distribution of the plantations is primarily concentrated in the western part of the country with the Bas-Sassandra, Montagnes and Sassandra-Marahoué districts having the highest area planted (Table 3, Fig. 5) and the greatest coverage. However, in terms of percentage coverage, the Gôh-Djiboua district (27.36%) has the highest values (Fig. 6). The relatively smaller districts (Yamoussoukro and Abidjan) have less coverage (20.1% and 12.6%, respectively), while these are the most economically advanced areas in the country. The high cocoa coverage areas in the western districts are situated where the last remaining forests in the region exist. This could indicate further young under canopy plantations, which might not have been detected.

A recent land cover and land use change monitoring dataset (Szantoi et al., 2020) indicated that the Tai-Sapo Key Landscape for Conservation area – which covers this geographical region of the country – lost most of its natural vegetation in the past 20 years (a part of the Tai National Park) to shrub crops (i.e. cocoa) and, moreover, demonstrated shrub crop encroachment into Liberia.

Ghana has around 2.15 Mha of cocoa plantations, and similarly to Côte d'Ivoire, these plantations are mainly concentrated in the western part of the country (Fig. 5): the Western (20.3%) and Ashanti (20.9%) regions and the Central region (22.1%) (Fig. 6). Two large regions, Brong-Ahafo (8.0%) and Volta (9.4%) have the least amount of cocoa coverage, which may be due to their geographical locations; Brong-Ahafo lies on the northern ‘edge’ of the cocoa belt, while Volta is also located in the northeastern part of the country. Interestingly, Ghana’s regions do not have more than 22% (Central) cocoa coverage, while in Côte d'Ivoire some districts have more than 25% cocoa coverage (e.g. Gôh-Djiboua, Montagnes, Sassandra-Marahoué) compared to other land cover/uses in those areas (Fig. 6).

### 3.3 Encroachment of cocoa plantations into and around protected areas (PAs)

Our results indicate that 1.18 Mha out of the 5.8 Mha of cocoa plantations we detected are located in PAs, and that cocoa plantations encroach into 362 distinct PAs – representing almost 70% of the PAs in the study area (Table 4).

**Table 4:** Characteristics of cocoa encroachment into protected areas* (PAs) by IUCN management category in terms of number of PAs affected (#), average cocoa area and average % of cocoa farms. *Protected areas with cocoa plantations present are calculated only.

IUCN Management Category	Côte d’Ivoire			Ghana			Ghana + Côte d’Ivoire		
	#	average cocoa area in PAs (ha)	average % of cocoa area in PAs	#	average cocoa area in PAs (ha)	average % of cocoa area in PAs	#	average cocoa area in PAs (ha)	average % of cocoa area in PAs
Ia	1	58.34	1.14	1	1442.39	3.87	2	750.36	3.54
II	7	2732.95	2.48	7	835.23	0.74	14	1784.09	1.60
III	0			47	1792.71	18.14	47	1792.71	18.14
IV	1	3855.82	13.77	62	1531.31	16.26	63	1568.21	16.15
V	0			5	1055.23	23.64	5	1055.23	23.64
VI	0			92	1405.23	18.43	92	1405.23	18.43
Not reported	144	5809.22	24.08	6	399.61	1.33	150	5592.84	22.96
Not Applicable	2	8001.05	3.01	1	39.23	0.13	3	5347.11	2.86
**Total**	**155**	**5648.87**	**18.21**	**221**	**1463.72**	**11.51**	**376**	**3188.98**	**15.74**

**Fig. 7:**
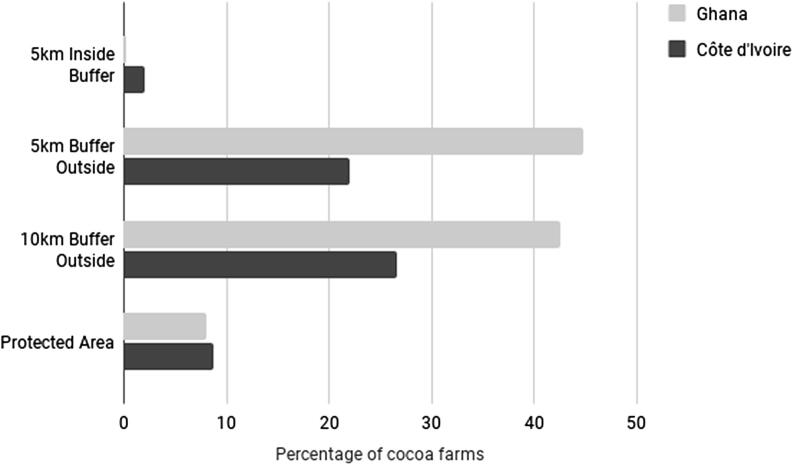
Percentage of cocoa area in protected areas and in 5 km and 10 km buffer areas outside the protected areas.

This phenomenon of cocoa encroachment into PAs is more pronounced for Côte d’Ivoire than for Ghana in terms of area (23% of the area of PAs are cocoa plantations in Côte d’Ivoire versus 14.5% in Ghana), but it is more pronounced for Ghana than for Côte d’Ivoire regarding the number of PAs (72% of PAs house cocoa plantations in Ghana versus 63% in Côte d’Ivoire) (Table 4, Fig. 7). The distribution of cocoa encroachment into PAs of known management categories (Table 4) indicates that, overall, cocoa encroachment is higher in PAs where the interaction of people and nature over time has produced an area of distinct character (management category V) than in PAs where the conservation of natural biodiversity is the main purpose (management category II) or those which are strict nature reserves (management category I). However, the management category is unreported for 40% of the PAs housing cocoa farms of the study area, and these PAs exhibit a high percentage of cocoa encroachment – the highest in Côte d’Ivoire (Table 4). Therefore, it is necessary to collect further information on the actual management of these PAs to determine whether and how such management explains the extent of cocoa encroachment into PAs.

Our findings support and take to an unprecedented level the worrying findings of Bitty et al. (2015) showing that cocoa plantations encroach into PAs. Furthermore, we show that cocoa encroachment into PAs is not limited to the periphery of PAs, since we detected cocoa plantations as far as 5 km inside of the external boundaries of PAs (Fig. 7). We also detected cocoa plantations in close proximity to PAs (Fig. 7). In Côte d’Ivoire, in particular, we observed an increasing percentage of cocoa area from PA to the 10 km outside buffer (Fig. 7), indicating that even if cocoa farms can be found in PAs, their concentration increases with the distance from the PA. The pattern is different for Ghana where, even if the concentration of cocoa farms is higher outside than inside PAs, cocoa farms are more concentrated within the 5 km outside buffer than the 10 km outside buffer (Fig. 7). The cocoa plantations detected in close proximity to PAs may correspond to the cocoa agroforestry systems which have been suggested as a management strategy to reduce the influence of surrounding land-use activity on PA biodiversity (Asare et al., 2014, Bitty et al., 2015). Although our study does not enable the direct investigation of how effective this strategy is in mitigating the effects of land-use activities on PA biodiversity, we clearly show that this strategy is ineffective in preventing the encroachment of cocoa plantations into PAs.

**Fig. 8:**
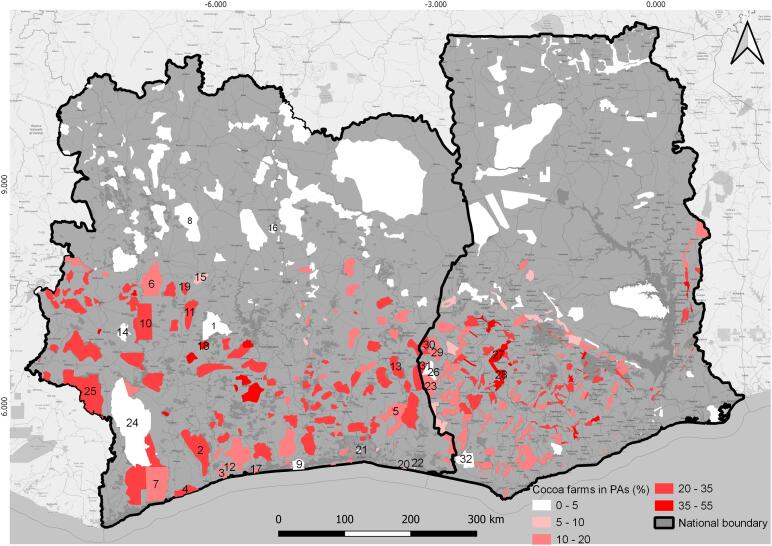
Estimated area of cocoa plantations (%) within protected areas. NP = National Park, FR = Forest Reserve, RR = Resource Reserve, NR = Not Reported. 1. Marahoué NP, 2. Niégré FR, 3. Dassieko FR, 4. Monogaga FR, 5.Mabi FR/Yaya FR, 6. Séguéla FR, 7. Rapide Grah FR, 8. Kani-Bandaman NR, 9. Azagny NP, 10. Sassandra FR, 11. Haut De, NR, 12. Bolo FR, 13. Bossematie FR, 14. Mont Peko NP, 15. Moyenne Marahoué NR, 16. Koba FR, 17. Port Gautier FR, 18. Bouafle FR, 19. De FR, 20. Ngadan Ngadan FR, 21. Banco NP, 22. Ile Ehotilé NP, 23. Bia Tawya FR, 24. Tai NP, 25. Anhia NR, 26. Bia NP, 27. Tano Ofin FR, 28. Asenanyo FR, 29. Bia North FR, 30. Manzan FR, 31. Sukusuki FR, 32. Ankasa RR.

The PAs housing cocoa plantations are concentrated in the southern parts of Côte d’Ivoire and Ghana, corresponding to the cocoa belt, and 105 PAs have cocoa plantations exceeding 35% of their area (Fig. 8).

**Fig. 9:**
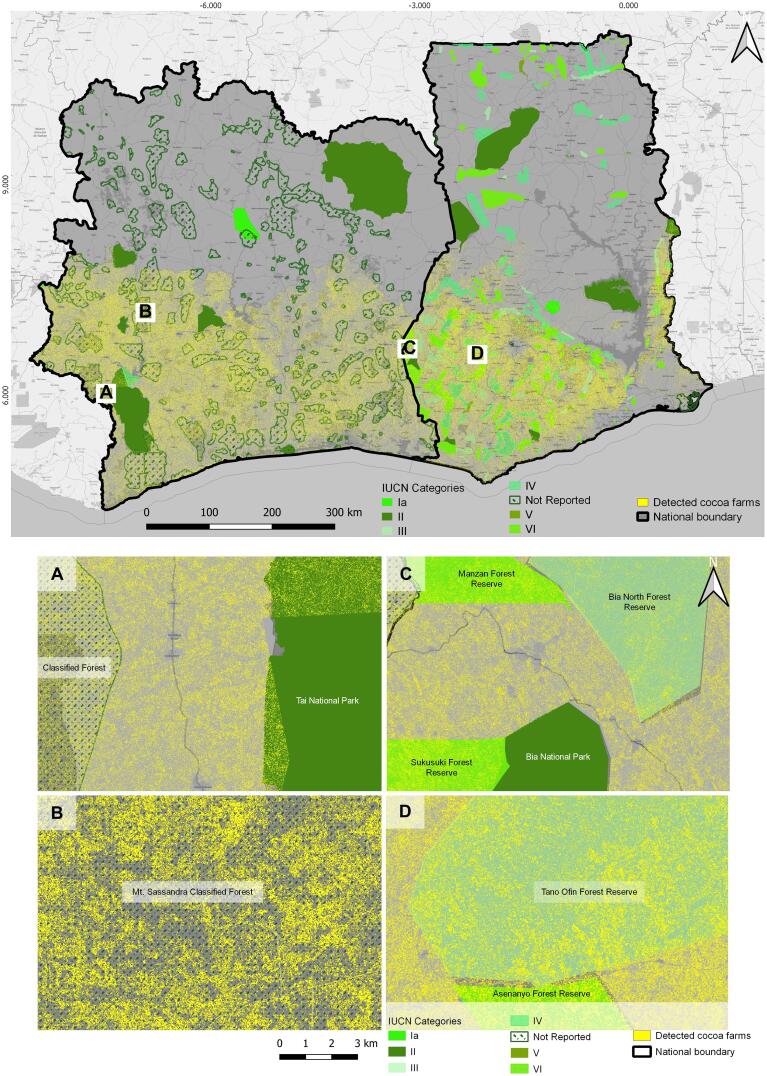
The top image is a detailed map showing the distribution of cocoa farms and protected areas (by management category) in Côte d’Ivoire and Ghana. The bottom images (A to D) are zooms in four locations. (A) Tai National Park (IUCN category II), free of cocoa inside but plantations are close to the boundary; (B) Mt Sassandra Forest Reserve (undesignated IUCN category) with a high density of cocoa farms; (C) Sukusuki (undesignated IUCN category) with a high density of cocoa farms and Bia Forest Reserve (IUCN category II) with no cocoa encroachment; (D) Anhwiaso East Forest Reserve (undesignated IUCN category) with a high density of cocoa farms.

In these southern areas, only a few PAs are free from cocoa plantations, such as the Tai Park in Côte d’Ivoire and Bia National Park and Ankasa Resource Reserve in Ghana (Fig. 8). However, even these few PAs free from cocoa plantations are directly surrounded by PAs and/or unprotected land with high concentrations of cocoa plantations (Fig. 9).

**Fig. 10:**
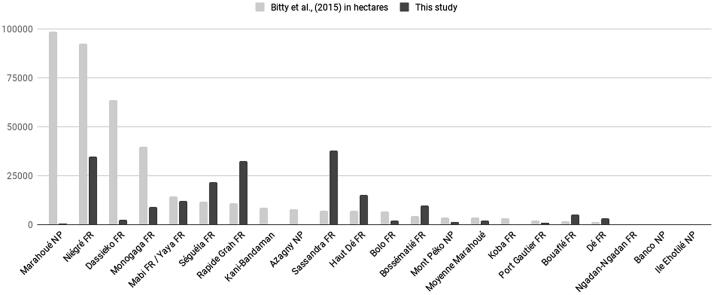
Area of cocoa farms (ha) in protected areas as estimated by Bitty and colleagues (Bitty et al., 2015) versus that estimated in this study. NP = National Park, FR = Forest Reserve. Mabi and Yaya FRs are treated as one in this study as they are reported as one FR in the WDPA.

Our results regarding the area of cocoa plantations in PAs are quite different from those estimated by Bitty and colleagues (2015). Indeed, although both studies agree on the fact that 3 out of the 23 PAs they surveyed are free of cocoa (Banco, Ile Ehotilé and Ngadan-Ngadan, Fig. 8, Fig. 10), we found that the total proportion of cocoa over these 23 PAs (13.2%) was smaller than that estimated by Bitty and colleagues (2015) (74%).

In detail, for 13 PAs Bitty and colleagues (2015) estimated a higher cocoa coverage than reported in the present study, while for 6 PAs it is the opposite, with −99.5% being the maximum difference between both estimates for the Maraouhé protected area (Fig. 10). These differences in estimates between studies could either be related to methodological differences (Bitty et al. carried out ground surveys while we used satellite imagery) or real differences regarding the location and extent of cocoa areas between 2010 and 2013 and 2020. If the latter explanation holds, the overall reduction in cocoa area in PAs we observed may be the result of the establishment of the Cocoa and Forests Initiative in 2017 (Mithöfer et al., 2017, Ruf and Varlet, 2017), which is an active commitment by top cocoa-producing countries with leading chocolate and cocoa companies to end deforestation and restore forest areas. However, only new ground surveys can be used to discriminate between these two explanations.

Overall, our findings highlight that deforestation is happening in PAs of Côte d’Ivoire and Ghana because of the conversion of primary forests into new cocoa plantations. There are a number of interrelated causes to explain this phenomenon which have been extensively explored in other studies (Asare et al., 2014, Ruf, 2011, Ruf and Varlet, 2017, Bymolt et al., 2018). They can be broadly classified into proximal causes (i.e. law and law enforcement is not strong enough to avoid cocoa plantations in PA) and more distal causes (once cocoa soils have lost their fertility, the only way to get a decent income for cocoa farmers is to expand their cocoa farms into newly deforested areas – rather than rehabilitating ageing cocoa farms). Although cocoa plantations in PAs are not strictly illegal (this depends on the management category of each PA), PAs cannot fulfil their role regarding forest conservation if a considerable portion of their area is occupied by cocoa plantations – as is the case in Côte d’Ivoire and Ghana. Fighting against cocoa-related deforestation would legally require protecting the remaining forest areas in PAs and ensuring that such legal protection is enforced. But this would also require fighting against more distal causes, for instance by reforming the current cocoa pricing system so that cocoa farmers make more money by rehabilitating old cocoa farms outside PAs rather than by creating new cocoa farms within PAs.

## 4 Conclusion and outlook

This is the first study to map the extent of cocoa farms at country level for Côte d'Ivoire and Ghana as derived from high spatial resolution satellite images using a consistent, repeatable approach. We observed a 1.51% difference in the cocoa area in Ghana and −0.97% in Côte d’Ivoire as compared to FAOSTAT (2018). Based on the results, three conclusions were drawn: (1) cocoa plantations can be mapped using freely available radar (Sentinel-1) and optical (Sentinel-2) imagery and their derivatives (vegetation indices, texture measures); (2) cocoa farms in Côte d’Ivoire and Ghana have a broad spatial distribution; (3) cocoa farms have largely encroached into PAs. This study reinforces previous findings (Bitty et al., 2015) showing that the current network of PAs in Côte d’Ivoire and Ghana is not preventing cocoa-related deforestation in such areas. These findings highlight the urgent need for governments and cocoa buyers to address both the distal and the proximal causes of cocoa-related deforestation. Other policy applications of the produced cocoa map include further environmental and socio-economic studies regarding the productivity, the quality and the sustainability of Ivorian and Ghanaian cocoa in both regions. This study also demonstrates a successful method to map cocoa farms at national level and shows potentials to be upscaled temporally and spatially. The producer’s and user’s accuracies of the generated cocoa map can be further improved by increasing the size of the training dataset, as well as employing deep learning algorithms such as semantic image segmentation.

## 5 Data accessibility

The complete cocoa map dataset is available from Abu et al. (2020) https://doi.pangaea.de/10.1594/PANGAEA.917473. The downloadable ‘MAIN.zip’ folder contains a GeoTiff dataset at 10 m spatial resolution. The cocoa maps are distributed through a dedicated website (https://land.copernicus.eu/global/hsm, last accessed: 28 January 2021), where users can visualise and download the data.

## CRediT authorship contribution statement

**Itohan-Osa Abu:** Writing - original draft, Writing - review & editing, Data curation, Methodology, Formal analysis, Investigation. **Zoltan Szantoi:** Conceptualization, Methodology, Writing - original draft, Formal analysis, Writing - review & editing, Investigation, Supervision. **Andreas Brink:** Conceptualization, Funding acquisition, Writing - original draft, Writing - review & editing, Investigation. **Marine Robuchon:** Writing - review & editing. **Michael Thiel:** Writing - original draft, Writing - review & editing.

## Declaration of Competing Interest

The authors declare that they have no known competing financial interests or personal relationships that could have appeared to influence the work reported in this paper.
